# Supplementary material for: Nomogram established on account of Lasso-Cox regression for predicting recurrence in patients with early-stage hepatocellular carcinoma
Source: Front Immunol. 2022 Nov 23;13:1019638. doi: 10.3389/fimmu.2022.1019638 (PMC9726717; doi:10.3389/fimmu.2022.1019638)
Supplement: Supplementary file 1 [file DataSheet_1.docx]

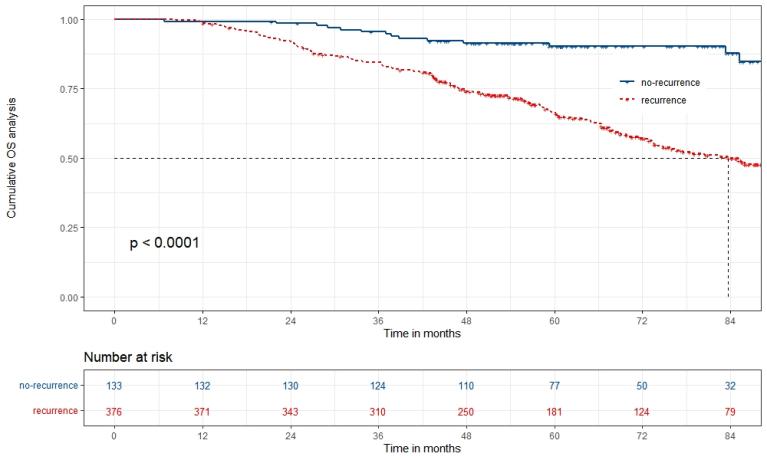


Fig.S1 The Kaplan-Meier analysis of OS for patients based on recurrence status.

Abbreviation: OS: overall survival.


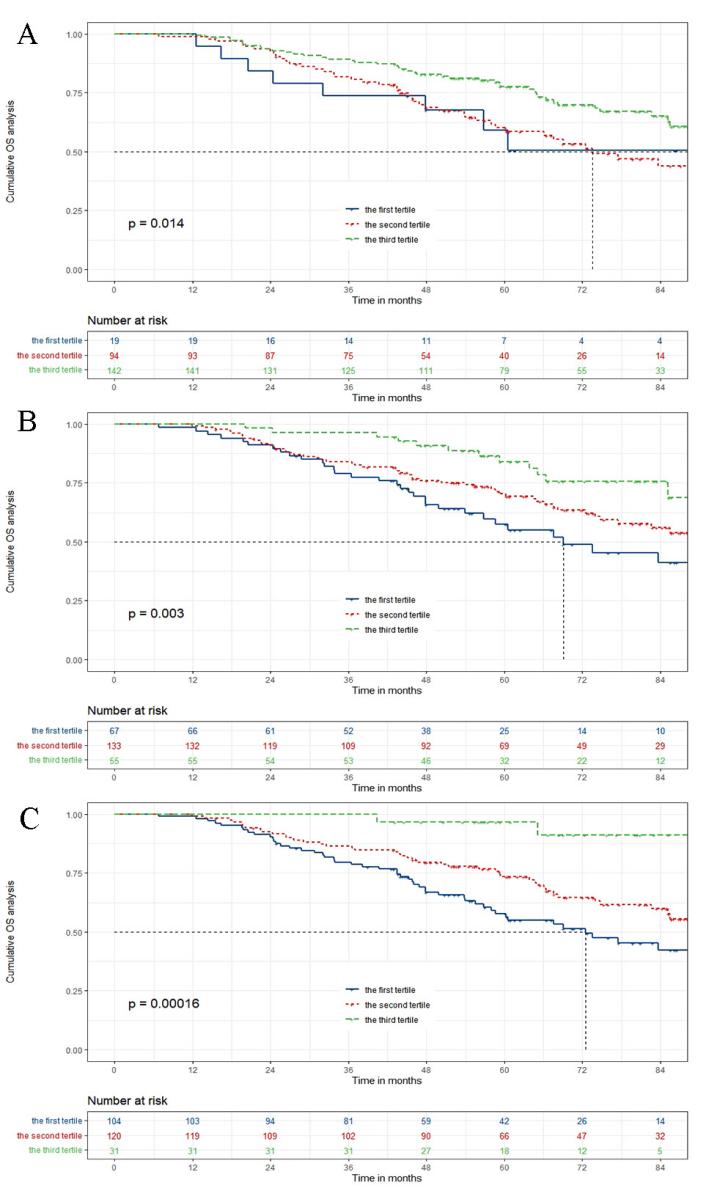


Fig.S2 The OS analysis by tertiles of predicted 1-, 3, and 5-year RFS according to Cox regression.

1. 1-year OS analysis; (B) 3-year OS analysis; (C) 5-year OS analysis.

Abbreviation: RFS: recurrence-free survival; OS: overall survival.
